# Ma xing shi gan decoction eliminates PM2.5-induced lung injury by reducing pulmonary cell apoptosis through Akt/mTOR/p70S6K pathway in rats

**DOI:** 10.1042/BSR20193738

**Published:** 2020-07-09

**Authors:** Yefang Wang, Bo Zhao, Yuxiang Fei, Qiyang Yin, Jianping Zhu, Guanghui Ren, Bowen Wang, Weirong Fang, Yunman Li

**Affiliations:** 1 Department of Paediatrics, Nanjing Integrated Traditional Chinese and Western Medicine Hospital, Nanjing 210014, People’s Republic of China; 2 State Key Laboratory of Natural Medicines, School of Basic Medicine and Clinical Pharmacy, China Pharmaceutical University, Nanjing 210009, People’s Republic of China

**Keywords:** Akt/mTOR/p70S6K pathway, apoptosis, lung injury, Ma xing shi gan Decoction, PM2.5

## Abstract

The present study was designed to investigate the anti-apoptosis effect of Ma xing shi gan decoction (MXD) on PM2.5-induced lung injury via protein kinase B (Akt)/mTOR/p70S6K pathway. A UPLC-MS/MS system was introduced for component analysis of MXD. Rats were instilled with PM2.5 solution suspension intratracheally to induce acute lung injury. The rats were then orally administered with MXD (16, 8, and 4 g/kg) once a day for 7 consecutive days. The therapeutic effects of MXD were evaluated by Hematoxylin and Eosin (HE) staining. The apoptotic cell death was analyzed by terminal-deoxynucleotidyl transferase-mediated nick-end labeling (TUNEL) assay. The alterations in cytochrome *c* (Cytc) and cleaved-caspase-3 (C-caspase-3) were measured by immunohistochemistry (IHC). The expressions of Bax, B-cell lymphoma 2 (Bcl-2), p-Akt, p-mTOR and p-p70S6K were detected by Western blot. *In vitro*, PM2.5 exposure model was introduced in A549 cell, followed by incubation with MXD-medicated serum. Hoechst staining was used to determine apoptotic rate. The levels of Bax, Bcl-2, p-Akt, p-mTOR and p-p70S6K were detected by Western blot. Our results *in vivo* indicated that treatment with MXD decreased histopathological changes score, TUNEL-positive cells rate, expressions of Cytc and C-caspase-3. The *in vitro* results revealed that incubation with MXD-mediated serum decreased apoptotic rate. Both results *in vivo* and *in vitro* demonstrated that MXD inhibited pro-apoptotic protein Bax and promoted anti-apoptotic protein Bcl-2 expression. Likewise, MXD activated Akt/mTOR/p70S6K signal pathway, which was also confirmed by Western immunoblotting. In conclusion, MXD attenuates lung injury and the underlying mechanisms may relate to regulating the apoptosis via Akt/mTOR/p70S6K signaling pathway activation.

## Introduction

Fine particulate matter (PM2.5) refers to particulate matter with an aerodynamic equivalent diameter of 2.5 microns or less in air. It can be suspended in the air for a long time, and the higher its concentration in the air, the more serious the air pollution will be [1]. Epidemiological studies demonstrate that PM2.5 exposure is closely related to respiratory diseases. Particles with a diameter of 10 μm are usually deposited in the upper respiratory tract, but particles with diameter less than 2.5 μm can penetrate deep into the bronchioles and alveoli. When PM2.5 enters the alveoli, it can directly affect the ventilation function of the lungs and cause lung injury [2]. With the development of industrialization, the concentration of PM2.5 in air is increasing year by year [3]. Long-term exposure to PM2.5 poses a great threat to human health, which has been confirmed by *in vivo* experiments. He et al. pointed out PM2.5 exposure causes oxidative stress-dependent inflammation in lungs which are rich in type II alveolar cells [4]. Meanwhile, a study suggested that PM2.5 induces cell apoptosis in human lung alveolar epithelial (A549) cells [5].

Apoptosis plays a crucial role in the pathogenesis and development of many respiratory diseases such as pneumonia and asthma, which is regulated by multiple pathways [6]. When PM2.5 stimulates the respiratory tract, PM2.5 detained in the bronchi and alveoli can cause damage or even death of the lung epithelial cells [7]. As the injury progresses, excessive apoptosis plays an important role in lung cell death, but the effective intervention remains to be explored.

With a long history and extensive clinical practice, the role of Chinese medicine in the treatment of some diseases attracted much attention [8]. Ma xing shi gan decoction (MXD) comprises the monarch drug Ephedra Herb, the minister drug Gypsum, the assistant drug Bitter Almond, and the guide drug Liquorice Root. As a classic famous prescription in Treatise on Febrile Diseases, it is able to treat lung diseases such as upper respiratory tract infection, acute bronchitis, pneumonia and bronchial asthma. It has shown an excellent safety and efficacy record for thousands of years. MXD has been reported to inhibit the expression of inflammatory factor expression in lung tissue induced by influenza virus A H1NI, and the terminal-deoxynucleotidyl transferase-mediated nick-end labeling (TUNEL) assay demonstrates the protection of MXD in lung tissue [9]. However, in recent years, few studies have reported the effect of MXD against PM2.5-induced lung injury at the perspective of anti-apoptosis.

The aim of the present study was to investigate the effect of MXD in attenuating PM2.5-induced lung injury and explore the underlying mechanism. Our results will provide a theoretical and practical basis for the treatment of PM2.5-induced lung injury.

## Materials and methods

### Experimental drugs and reagents

PM2.5 collected from January to May 2018 in suburb of Nanjing was kindly provided by Nanjing Environmental Monitoring Center. After sterilization under ultraviolet radiation for 1 h, the concentration of PM2.5 suspension was adjusted to 10 mg/ml with deionized water. Before use, the suspension was fully suspended to avoid agglomeration.

All crude drugs were purchased by the pharmacy of Nanjing Integrated Traditional Chinese and Western Medicine Hospital and identified as authentic by Yong Wang (Professor, Nanjing Integrated Traditional Chinese and Western Medicine Hospital, China).

The composition and doses of MXD have been listed in Table 1. Ephedra Herb (Sanyue Traditional Chinese Medicine Co., Ltd, Nantong, China) was decocted in 1 l of double distilled water for 1 h. After skimming the scum, Bitter Almond (Jiangsu Huahong Pharmaceutical Technology Co., Ltd, Danyang, China), Gypsum (Jiangsu Huahong Pharmaceutical Technology Co., Ltd, Danyang, China) and Liquorice Root (Hangzhou Zhende Traditional Chinese Medicine Co., Ltd, Hangzhou, China) were added and boiled for 40 min. After that the extract was filtered and enriched by rotary evaporation. The crude drug concentration of MXD was 2 g/ml. For drug administration at different doses, MXD was diluted using normal saline.

**Table 1:** The composition of MXD

English name	Latin name	Chinese name	Proportion (g)
Ephedra Herb	Ephedra sinica Stapf	Ma huang	4
Bitter Almond	Armeniacae Amarum Semen	Ku xing ren	12
Gypsum	Gypsum fibrosum	Shi gao	24
Liquorice Root	Glycyrrhiza uralensis Fisch	Gan cao	8

### Quantitative analysis of MXD by ultra-performance liquid chromatography-tandem mass spectrometry/mass spectrometry

Standards of Ephedrine hydrochloride (CAS No. 50-98-6, purity 100.0%) and pseudoephedrine hydrochloride (CAS No. 345-78-8, purity 99.8%) were purchased from National Institutes for Food and Drug Control. Standards of Liquiritin (CAS No. 551-15-5, purity ≥ 98%), Glycyrrhizic acid ammonium salt (CAS No. 53956-04-0, purity ≥ 98%) and Amygdalin (CAS No. 29883-15-6, purity ≥ 98%) were purchased from Solarbio Science and Technology Co., Ltd., Beijing, China.

The main chemical components of MXD were revealed by ultra-performance liquid chromatography-tandem mass spectrometry/mass spectrometry (UPLC-MS/MS). MXD was diluted 10000-times with 50% (v/v) methanol in water. The test solution was obtained after filtration.

The reference standard samples (ephedrine hydrochloride, pseudoephedrine hydrochloride, liquiritin, glycyrrhizic acid ammonium salt and amygdalin) were weighed accurately and dissolved in 50% (v/v) methanol in water at a concentration of 1 mg/ml. After diluting and pooling, the mixed reference standard solution containing ephedrine hydrochloride (500 ng/ml), pseudoephedrine hydrochloride (500 ng/ml), liquiritin (500 ng/ml), glycyrrhizic acid ammonium salt (500 ng/ml) and amygdalin (500 ng/ml) was obtained.

The preliminary analysis was performed using an Acquity UPLC BEH C18 (2.1 × 50 mm, 1.7 μm) column in both positive and negative electrospray ionization mode. For positive electrospray ionization mode, mobile phase solvent A consisted of aqueous phase of ammonium acetate (5 mmol/l), and mobile phase solvent B was pure methanol. The common elution condition is shown in Table 2.

**Table 2:** Elution condition for preliminary UPLC-MS analysis

Time (min)	MP A%	MP B%	Flow rate (ml/min)
Initial	98.0	2.0	
2.00	98.0	2.0	
2.40	70.0	30.0	
8.00	5.0	95.0	0.400
9.00	5.0	95.0	
9.20	98.0	2.0	
10.00	98.0	2.0	

### Animals and grouping

Male Sprague–Dawley rats (200–250 g) were purchased from Qinglongshan Animal Farm of Nanjing (Nanjing, China, license number: SCXK (Su) 2019-0001). All the experiments took place in the China Pharmaceutical University (Nanjing, China). Animals were used after 7-day acclimation. All animals were maintained under standard environment conditions (23 ± 2°C, 55 ± 5% humidity and 12-h/12-h light/dark cycle). All animals were allowed free access to tap water and standard laboratory rat food. All animal studies were in accordance with the National Institute of Health (NIH) guidelines for the Care and Use of Laboratory Animals approved by the Animal Ethics Committee of China Pharmaceutical University. All efforts were made to minimize animal suffering.

Rats were randomly divided into six groups: control group, intervention group, PM2.5-stimulated group, MXD (16 g/kg) group, MXD (8 g/kg) group, MXD (4 g/kg) group. In order to verify whether MXD interferes with normal rats, an intervention group was introduced. The middle dose of MXD was chosen on the basis of human clinical dose (1.3 g/kg) × 6.3 (conversion coefficient between rat and human) [10]. Two hours after the last PM2.5 suspension instillation, rats were intragastrically administered with MXD in different dosages at the volume of 1 ml/100 g for 7 consecutive days. Rats in intervention group were treated with MXD at the dose of 16 g/kg. Rats in control group and PM2.5-stimulated group were administered with the same volume of normal saline.

### Surgical procedure of PM2.5-induced model and drug treatment

Rats were anesthetized with 3% isoflurane in O_2_ for 40 min with a respirator mask, fixed in the supine position and tilted to an angle of 45° to horizontal plane. Then the fixed device was placed on a shaking table (vacillating to the left and right). Acute lung injury was then induced by PM2.5 instillation. Briefly, the rat’s trachea was inserted by a trocar (0.6 mm in inner diameter), followed by PM2.5 suspension injection into the trachea through the trocar at a volume of 0.1 ml/100 g body weight. Then the shaking table was turned on to rolled rats gently 60 s (at the speed of 30 cycles per minute) for distributing PM2.5 suspension equally in both lungs. This operation was done at the first, third, fifth and seventh days. Two hours after the last instillation of PM2.5 suspension, MXD was administered as described in section “Animals and grouping”. Rats in control group received tracheal intubation except for PM2.5 instillation.

### Tissue preparation

Two hours after the last administration, rats were killed by intravenous injection of pentobarbital (100 mg/kg), followed by perfusion with 50 ml normal saline, after which lungs were rapidly removed and fixed in 4% paraformaldehyde for 24 h. The lungs were then immersed in a 30% sucrose solution in PBS and stored at 4°C until section cutting. Lungs were embedded in paraffin and sliced into sections with 4-micron-thickness.

### Hematoxylin and Eosin staining

Sections of paraffin-embedded tissue were subjected to Hematoxylin and Eosin (HE) staining to estimate damage situation of alveolar and peribronchial lesions as described previously [11]. The histopathological changes of lung tissues were then observed with the optical microscope (Olympus BX53; Japan). The degree of lung injury was graded on a scale of 0–4 (0, absent; 1, light; 2, moderate; 3, strong; 4, intense) which was based on alveolar septal thickening, proteinaceous debris, neutrophils infiltration and other histologic features [12].

### Immunohistochemistry

The paraffin sections were subjected to immunohistochemical staining to quantify the expression of Cytochrome *c* (Cytc) and cleaved-caspase-3 (C-caspase-3), two main effector proteins during apoptosis. The sections were blocked with 10% normal goat serum, and incubated overnight with primary antibody to Cytc (Servicebio, China, 1:1000) and C-caspase-3 (servicebio, China, 1:1000) at 4°C. After washing with PBS for three times, sections were exposed to the secondary antibody at 37°C for 20 min, and then visualized using 0.05% 3,3′-diaminobenzidine-solution (DAB) [13]. The signal intensity of immunoreactivity was semiquantitatively evaluated using H-score analysis according to a previous study. The sections were then counterstained using Hematoxylin, rinsed with 1% hydrochloric acid alcohol, cleared in xylene and mounted. The expression of Cytc and C-caspase-3 was evaluated using the optical microscope in areas chosen randomly. The results were analyzed using ImageJ 1.50i (National Institutes of Health, U.S.A.).

### TUNEL assay

To examine the protection of MXD on apoptotic cell death after exposure to PM2.5, TUNEL assay was conducted according to previously published methods [14]. The paraffin sections were incubated with the TUNEL assay kit reagents for 1 h at 37°C after blockage and permeabilization. Sections were counterstained with DAPI. The slides were photographed using fluorescence microscope (Nikon Eclipse Ti-SR; Japan) and the images were analyzed using ImageJ 1.50i.

### Preparation of MXD-medicated serum

Five rats (250–280 g) were intragastrically administrated with MXD at 16g/kg for 7 days. Then blood samples were obtained from the arteria carotis communis, followed by centrifugation at 3000×***g*** for 10 min and inactivation at 56°C water bath for 30 min. Then the serum was filtered through a filter having a pore size of 0.22 microns, pooled and stored at −80°C for further use. Another five rats were administered with equal volume of normal saline, the nonimmune serum was collected and handled as described above, and used as control serum. The MXD-medicated serum was diluted by Dulbecco’s modified Eagle’s medium (DMEM) at various concentrations (volume fractions): 20% medicated serum; 10% medicated serum + 10% nonimmune serum; 5% medicated serum + 15% nonimmune serum. The quantification of main ingredients of interest in the medicated serum are shown in Supplementary Materials.

### Cell culture and PM2.5 stimulation

A549 cells, a human alveolar basal epithelial cell line, was purchased from American Type Culture Collection (ATCC) and cultured in DMEM with 10% fetal bovine serum, 5% CO_2_ and 95% O_2_ at 37°C. The cells were digested and passaged every 1 or 2 days.

Cells were seeded in 96-well plates at the density of 5 × 10^3^ cells/well and cultured overnight. Then, cells were randomly divided into six groups: control group, intervention group, PM2.5-exposed group and three treatment groups. The A549 cells in control group were incubated with nonimmune serum 20% for 24 h. Cells in intervention group were incubated with MXD-medicated serum at the volume fraction of 20% for 24 h. Cells in PM2.5 group were stimulated with PM2.5 suspension at the concentration of 20 μg/cm^2^ for 24 h. Cells in the treatment groups were incubated with various concentrations (volume fractions) of MXD-medicated serum (20% medicated serum; 10% medicated serum + 10% nonimmune serum; 5% medicated serum + 15% nonimmune serum) right after the addition of PM2.5 for 24 h. The PM2.5 concentration and incubation time were determined by previous study (data not shown). The total volume fractions of serum in all groups were strictly adjusted to 20% for uniformity.

### Cell viability assay

Cells were incubated with different concentrations (20, 10, 5%) of MXD-medicated serum to assess the cytotoxicity of MXD-medicated serum. To preliminarily evaluate the protection of MXD-medicated serum against PM2.5 challenge, 24 h after the incubation of medicated serum and PM2.5, 20 μl MTT solution (5 mg/ml) was added to each well followed by incubation for another 4 h at 37°C in darkness. Supernatant of medium containing MTT was carefully removed, and the black-purple crystals were dissolved by 200 μl DMSO. After a 10-min shaking, the absorbance was measured at 570 nm using a microplate reader (Thermo Multiskan FC, U.S.A.).

### Hoechst staining

The apoptotic cells were analyzed by Hoechst staining as previously described [15]. Briefly, 24 h after the induction of MXD-medicated serum and PM2.5, cells were incubated with Hoechst 33258 (Wanleibio, China) at room temperature for 10 min. After washing with PBS for three times (5 min per time), the images were captured under a fluorescence microscope (Olympus IX53; Japan).

### Western blot

Lung tissues and cells were lysed conventionally using the RIPA lysis buffer containing a mixture of PMSF and phosphatase inhibitor (v/v = 97:1:2) separately on ice. The total protein concentration was determined by bicinchoninic acid (BCA) protein assay. Equal amount of proteins was subjected to 10% SDS/PAGE and transferred on to polyvinylidene fluoride (PVDF) membranes [16]. After the transfer, the blots were blocked with 5% skimmed milk for 2 h and incubated overnight with diluted primary antibody B-cell lymphoma 2 (Bcl-2; Wanleibio, 1:500), Bax (Abways, 1:1000), β-actin (Abways, China, 1:3000), protein kinase B (Akt/PKB; Wanleibio, 1:500), phospho-Akt (Ser^473^, Wanleibio, 1:500), phospho-mTOR (Ser^2448^, Wanleibio, 1:500), mTOR (Wanleibio, 1:500), phospho-p70S6k (Thr^389^, Cell Signaling Technology, 1:500), p70S6k (Cell Signaling Technology, 1:1000). After washing with PBS (10 min × 3-times), bands were incubated with horseradish peroxidase (HRP)-conjugated anti-rabbit IgG (Abways, China, 1:5000) for 2 h at room temperature. The blots were then developed using enhanced chemiluminescence (ECL) solution (Applygen Technologies Inc.). The semi-quantitative analysis was performed using densitometry analysis by ImageJ 1.50i.

### Statistical analysis

All data are expressed as means ± S.D. Statistical analysis was performed using SPSS 22.0 statistical software (IBM Corp, U.S.A.) by one-way analysis of variance (ANOVA), followed by LSD post-hoc test (homogeneity of variance) and Games–Howell post-hoc test (heterogeneity of variance). A value of *P*<0.05 was considered as statistically significant.

## Results

### Chemical standardization of MXD by UPLC-MS/MS

**Figure 1:**
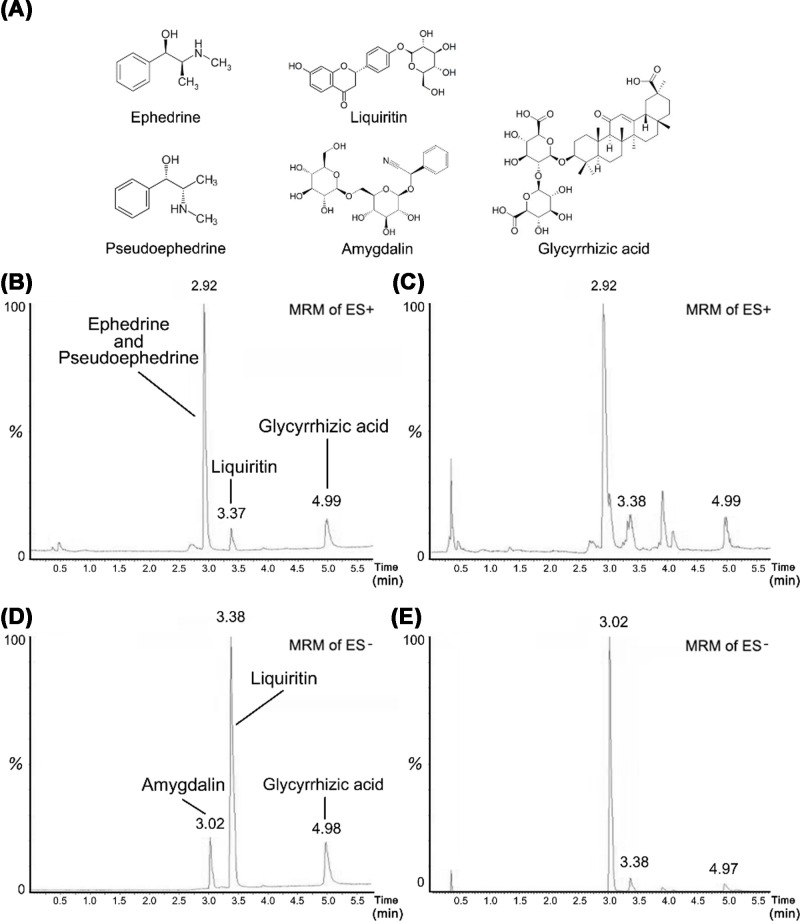
Chemical structures of the five major components in MXD and representative chromatograms of mixed standard solution and sample (**A**) Chemical structures of the five major components in MXD. Representative chromatograms of standard solution (**B**) and MXD sample (**C**) in positive electrospray ionization mode. Representative chromatograms of standard solution (**D**) and MXD sample (**E**) in negative electrospray ionization mode. The corresponding relation between chromatographic peaks and compounds are shown in the figures.

The MXD was analyzed using UPLC–MS/MS to quantify the main ingredients and obtain the chemical profile. Mass spectrometry was acquired in positive and negative electrospray ionization modes. Five major components (Figure 1A) were identified in MXD sample. A positive electrospray ionization mode LC-MS profile was developed for MXD sample and mixed standard solution (Figure 1B,C), and the result confirmed the presence of ephedrine hydrochloride, pseudoephedrine hydrochloride, liquiritin and glycyrrhizic acid in MXD. Besides, a negative electrospray ionization mode LC-MS profile was developed for MXD sample and mixed standard solution (Figure 1D,E), and the result confirmed the presence of amygdalin, liquiritin and glycyrrhizic acid in MXD.

### Therapeutic effects of MXD on lung injury in PM2.5-stimulated rats

**Figure 2:**
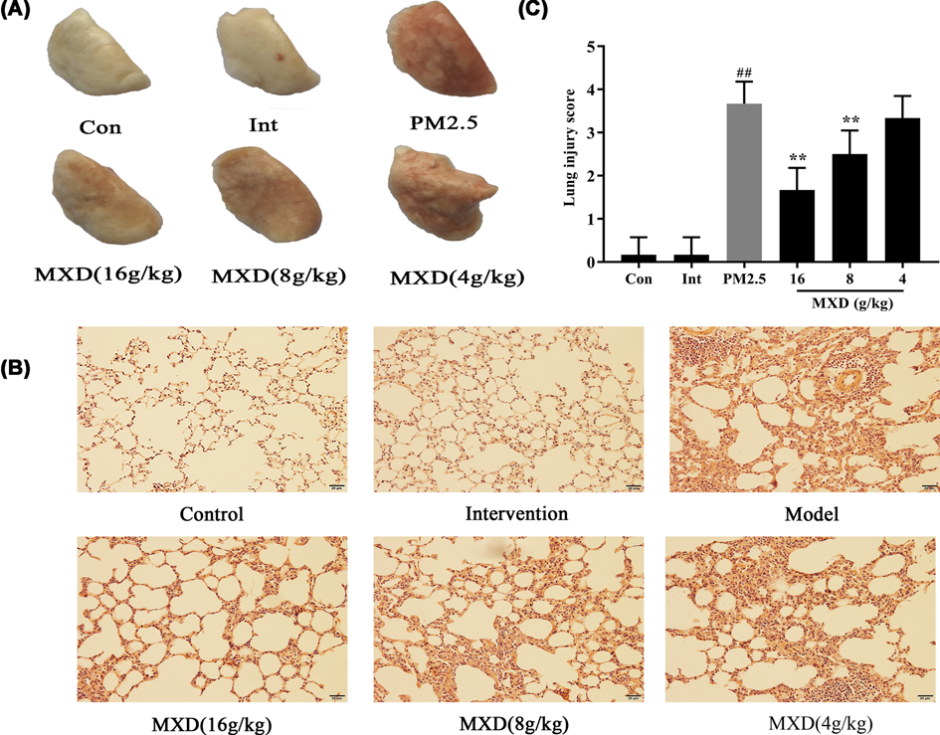
Therapeutic effect of MXD (16, 8, 4 g/kg) against PM2.5-induced lung injury after treatment with MXD for 7 consequtive days The PM2.5-stimulated group, MXD treatment group underwent PM2.5 stimulation while the control group and intervention group underwent the same surgical procedure without the transtracheal instillation. (**A**) Observation of pathological changes in lung tissues by naked eye after normal saline infusion. (**B**) Representative HE staining images of rat lung tissues (magnification, ×200). (**C**) Lung histologic injury score. Data are shown as mean ± S.D. *n*=6/group, **P<0.01 compared with PM2.5-stimulated group; ^##^*P*<0.01 PM2.5-stimulated group compared with control group. Con, control group; Int, intervention group; PM2.5, PM2.5-stimulated group.

Acute lung injury model was established in rats to investigate the protective effects of MXD against PM2.5-induced lung injury. The lungs of rats exposed to PM2.5 exhibited a significant degree of damage with obvious swell, congestion and blood spots (Figure 2A). Significant pathological changes, including alveolar wall thickening, pulmonary interstitial edema and inflammatory cell infiltration were observed in the lung tissues of PM2.5-stimulated rats (Figure 2B). Histological examination showed that lung injury score in PM2.5-stimulated group was markedly increased compared with the control group and intervention group (*P*<0.01). Treatment with MXD (16 and 8 g/kg) for 7 consecutive days significantly attenuated the histopathological changes induced by PM2.5 (Figure 2A,B). In addition, damage scores in the 16 and 8 g/kg MXD groups were significantly lower than that in PM2.5-stimulated group (*P*<0.01, Figure 2C). These results preliminarily demonstrated that treatment with MXD attenuates lung injury in PM2.5-stimulated rats.

### MXD treatment inhibits pulmonary cell apoptosis in PM2.5-stimulated rats

**Figure 3:**
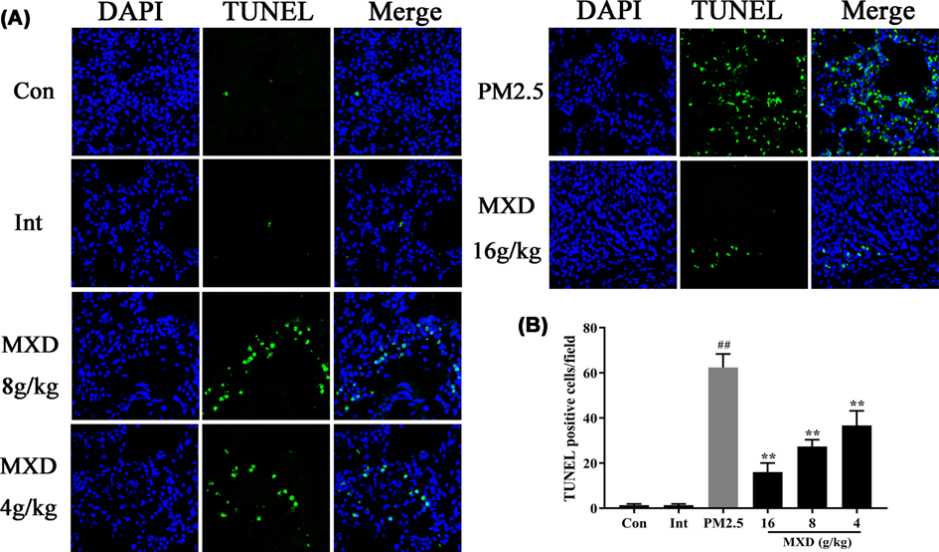
The effect of MXD on the number of TUNEL-positive cells in rat lung tissues after treatment with MXD for 7 consecutive days (**A**) Representative images of TUNEL staining, TUNEL-positive staining (in green) and DAPI (in blue) of the rat lung tissues. TUNEL-positive cells in five fields were counted under ×400 magnification. (**B**) Quantitative analysis of TUNEL positive cells. Data are shown as mean ± S.D. *n*=3/group, **P<0.01 compared with PM2.5-stimulated group; ^##^*P*<0.01 compared with control group.

For investigating the effects of MXD on pulmonary cell apoptosis after PM2.5 challenge, TUNEL assay was conducted in rat lung tissues. TUNEL-positive cells increased significantly in PM2.5-stimulated group compared with the control group and intervention group (*P*<0.01). Notably, compared with the PM2.5-stimulated group, the apoptosis rate of MXD-treated groups decreased significantly (*P*<0.01), demonstrating that MXD has the ability to prevent pulmonary cell apoptosis after PM2.5 challenge (Figure 3).

### MXD down-regulated Cytc and C-caspase-3 expressions in PM2.5-stimulated rats

**Figure 4:**
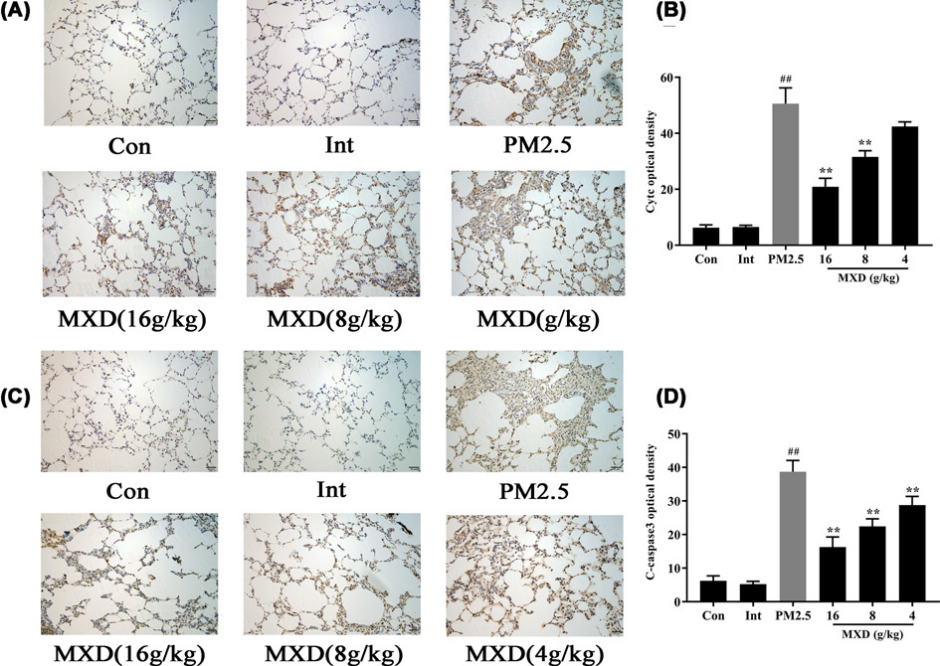
The effect of MXD on the expression of Cytc and C-caspase-3 in rat lung tissues after treatment for 7 consecutive days (**A**) Representative images of immunohistochemical staining (magnification, ×200) of Cytc in rat lungs. (**B**) Quantification of the positive staining (integrated optical density) of Cytc. (**C**) Representative images of immunohistochemical staining (magnification, ×200) of C-caspase-3 in rat lungs. (**D**) Quantification of the positive staining (integrated optical density) of C-caspase-3. Each column represents the mean ± S.D., *n*=3/group, **P<0.01 compared with PM2.5-stimulated group; ^##^*P*<0.01 compared with control group.

Cytc and C-caspase-3 are critical proteins involved in the process of apoptosis, and abnormal elevation of Cytc level may activate the caspase-3, which acts as an executioner during apoptosis. To perceive the expression of Cytc and C-caspase-3, immunohistochemistry (IHC) staining analysis was conducted after a 7-day MXD treatment. As shown in Figure 4, challenge of PM2.5 significantly increased the expression of Cytc (*P*<0.01) and C-caspase-3 (*P*<0.01) compared with the control group and intervention group. Notably, MXD (8 and 16 g/kg) suppressed the expression of Cytc (*P*<0.01) and C-caspase-3 (*P*<0.01), indicating MXD could inhibit the activation of Cytc and C-caspase-3 significantly.

### MXD protected cell viability when A549 cells exposed to PM2.5

First, the viability of A549 was measured by MTT assay to explored the toxicity of MXD under our laboratory conditions. Compared with the control group, the viability of A549 in MXD-medicated serum at concentrations 20, 10, 5 and 2.5% exhibited no significant difference (*P*>0.05). Therefore, the high concentration of MXD medicated serum was set at 20%, and the low concentration at 5%. To quantify the main ingredient in the-medicated serum, the representative chromatograms of the five compounds in reference standard solution and medicated serum were shown in Supplementary Figure S1.

**Figure 5:**
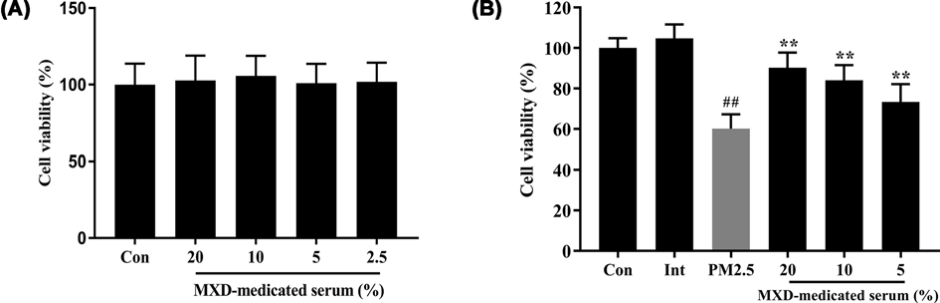
Effect of MXD-medicated serum on the cell viability of A549 cells after a 24-h incubation (**A**) The cytotoxic effects of different concentrations of MXD-medicated serum. (**B**) Except the control group and intervention group, the PM2.5-stimulated group, MXD group were treated with the intervention of PM2.5. Data are shown as mean ± S.D. *n*=8/group, **P<0.01 compared with PM2.5-stimulated group; ^##^*P*<0.01 compared with control group.

The viability of A549 cells was measured by MTT assay for preliminarily evaluating the protection of MXD-medicated serum against PM2.5 challenge. As shown in Figure 5, after PM2.5 exposure, the cell viability of A549 cells was strikingly decreased in comparison with control group and intervention group (*P*<0.01). However, after incubation with different concentrations of MXD-medicated serum, the viability of A549 cells was remarkably enhanced in comparison with PM2.5-stimulated group in a dose-dependent manner.

### MXD treatment inhibits cell apoptosis when A549 cells exposed to PM2.5

**Figure 6:**
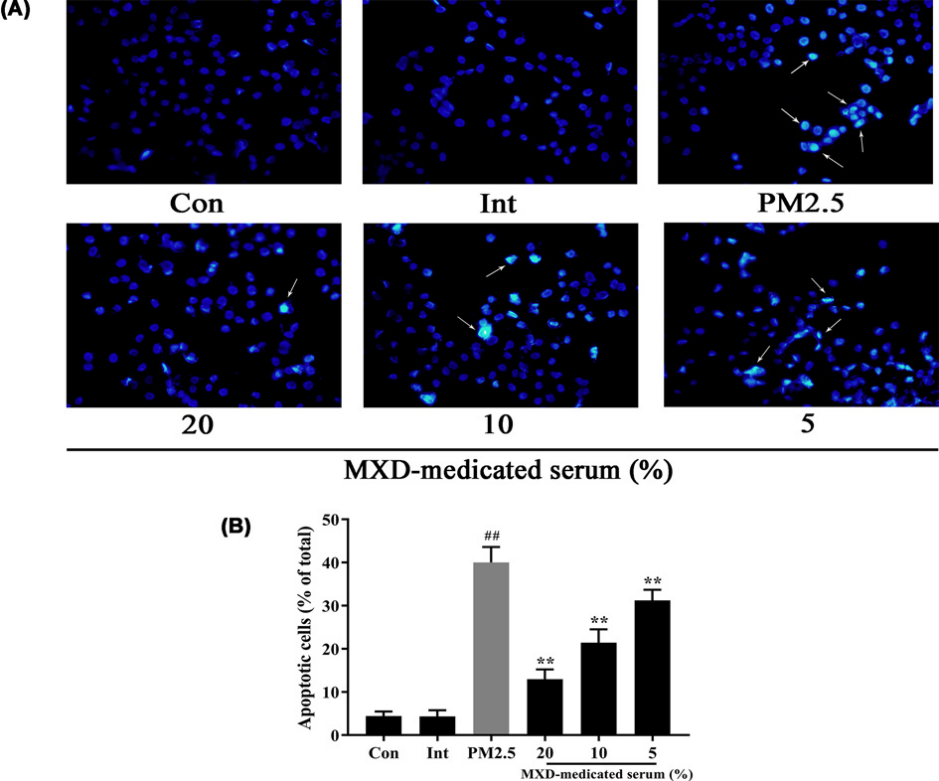
MXD-medicated serum protects against PM2.5-induced cell apoptosis in A549 cells (**A**) Changes in nuclear morphology of A549 cells were detected by Hoechst 33253 staining (magnification, ×200). The arrows represent apoptotic cells. (**B**) Quantitative analysis of the number of apoptotic cells. Data are shown as mean ± S.D. *n*=6/group, **P<0.01 compared with PM2.5-stimulated group; ^##^*P*<0.01 compared with control group.

Hoechst 33253 has the ability to stain the nucleus of the cells and normally applied to assess the level of apoptosis based on the specific morphological changes in nucleus during apoptosis [17]. The bright spots which indicate nuclear condensation can be observed in apoptotic cells after the exposure to PM2.5 for 24 h. However, there were no obvious apoptotic changes observed in the control group and intervention group. After incubation of MXD-medicated serum with different concentrations, the apoptotic rate was decreased significantly compared with the PM2.5-stimulated group (*P*<0.01, Figure 6).

### MXD regulated Bax and Bcl-2 expressions *in vitro* and *in vivo*

**Figure 7:**
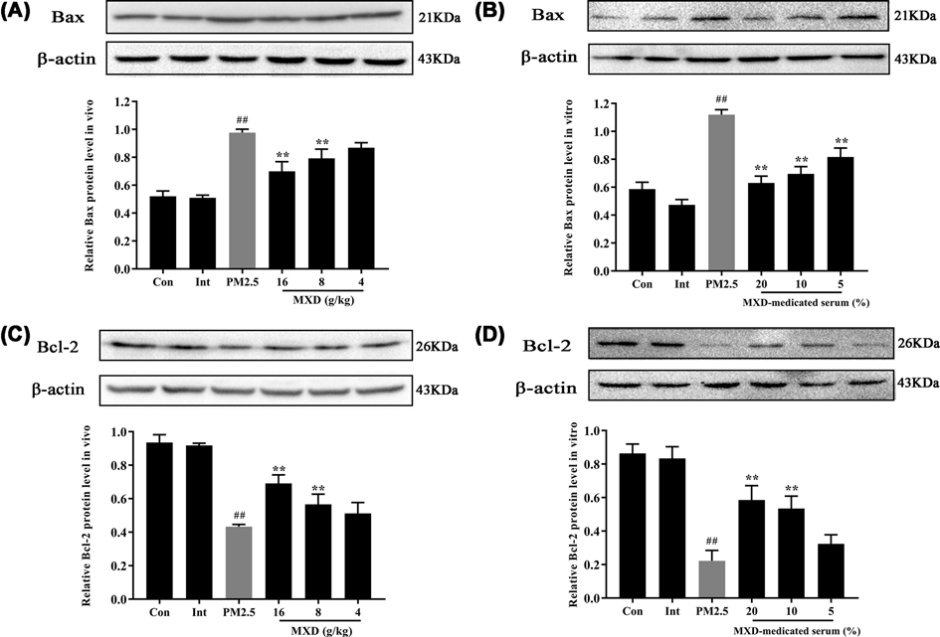
Effect of MXD (or MXD-medicated serum) on Bcl-2 and Bax protein expression in PM2.5-stimulated rats lung tissue and A549 cells (**A**) Bax protein expression *in vivo*. (**B**) Bax protein expression *in vitro*. (**C**) Bcl-2 protein expression *in vivo*. (**D**) Bcl-2 protein expression *in vitro*. Data are shown as mean ± S.D. *n*=3/group, **P<0.01 compared with PM2.5-stimulated group; ^##^*P*<0.01 compared with control group.

The expression of Bax and Bcl-2 were further investigated both *in vivo* and *in vitro* by Western blot. As shown in Figure 7A,B, compared with the control group, PM2.5 exposure induced a significant increase in the expression of Bax and decrease in the expression of Bcl-2, *in vivo* and *in vitro* (*P*<0.01). However, treatment with MXD (16, 8 g/kg) and MXD-medicated serum (20 and 10%) significantly lowered the expression of Bax (*P*<0.01). Besides, the expression of Bcl-2 was significantly increased by MXD (16, 8 g/kg) and MXD-medicated serum (20 and 10%) in comparison with PM2.5 group (*P*<0.01, Figure 7C,D).

### MXD regulated the activation of Akt/mTOR/p70S6K pathway *in vivo* and *in vitro*

**Figure 8:**
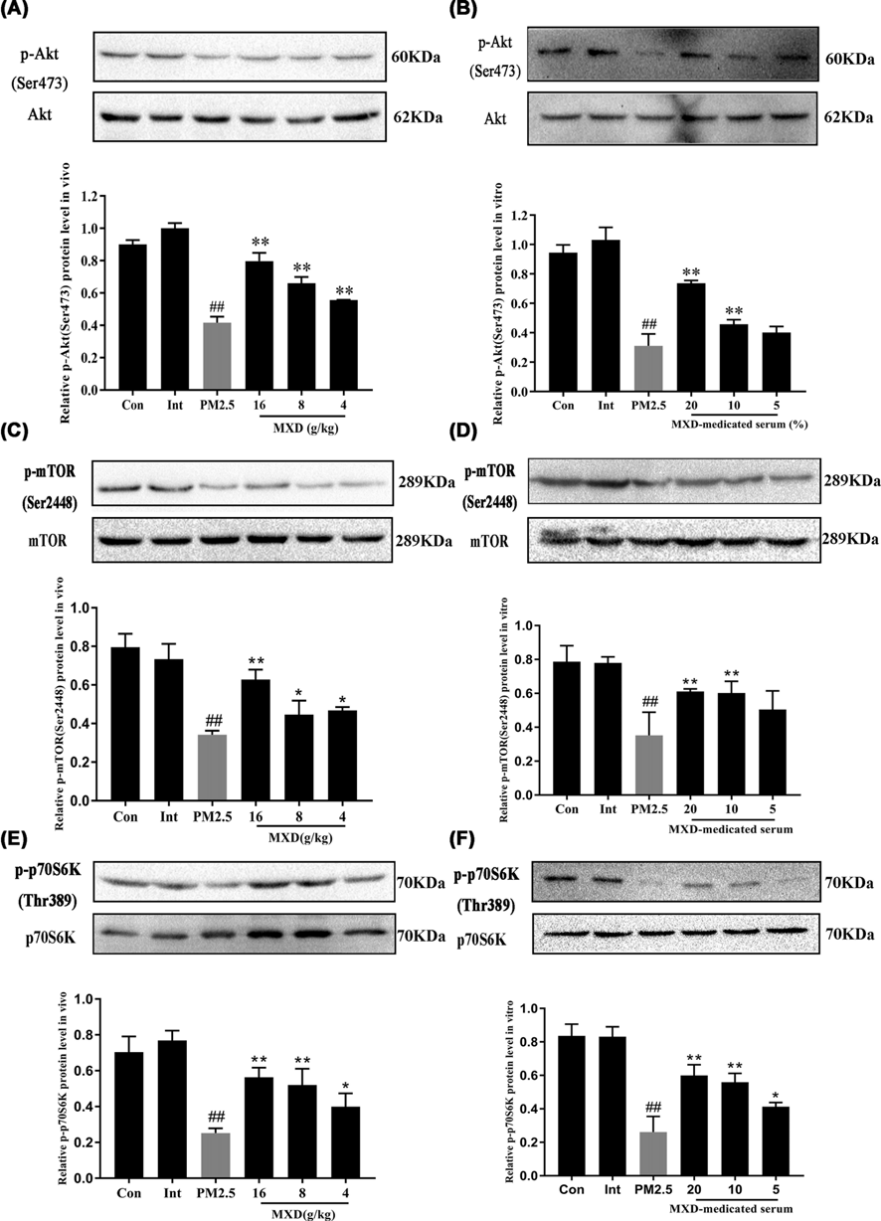
Effect of MXD (or MXD-medicated serum) on phosphorylated Akt, mTOR, P70S6K expressions in PM2.5-stimulated rat lung tissues and A549 cells (**A**) *In vivo* p-Akt expression. (**B**) *In vitro* p-Akt expression. (**C**) *In vivo* p-mTOR expression. (**D**) *In vitro* p-mTOR expression. (**E**) *In vivo* p-p70S6K expression. (**F**) *In vitro* p-p70S6K expression. Data are expressed as mean ± S.D. *n*=3/group, *P<0.05, **P<0.01 compared with PM2.5-stimulated group; ^##^*P*<0.01 compared with control group.

For further confirming the molecular pathway mediating the anti-apoptotic effect of MXD, the effect of MXD on the activation of Akt/mTORC1/p70S6K was examined by Western blot both *in vivo* and *in vitro*. As shown in Figure 8, in the PM2.5-stimulated group, the expression of the phosphorylated Akt was significantly down-regulated compared with control group (*P*<0.01). After treatment with MXD (16 g/kg) *in vivo*, the expression of p-Akt, p-mTOR and p-p70S6K were increased significantly compared with PM2.5-stimulated group (*P*<0.01). For *in vitro* assay, treatment with MXD-medicated serum (20 and 10%) significantly increased the phosphorylation of Akt, p-mTOR and p-p70S6K (*P*<0.01) which was in accordance with the results *in vivo*.

## Discussion

In the current study, we showed clearly that MXD could eliminate PM2.5 stimulation-induced apoptosis via modulating ratio of Bax/Bcl-2 and up-regulate expression of Akt in a dose-dependent manner both *in vivo* and *in vitro*. The underlying mechanisms appear to be attributable to the activation of the Akt signaling pathway.

MXD has been used to treat lung diseases since ancient times. The clear pharmacological effects of the four herbal medicines in MXD have been confirmed. The pseudoephedrine/ephedrine in Ephedra Herb inhibited the apoptosis of hepatocyte [18]. Liquiritin, a major active component of licorice root, protects mice and HACAT cells from solar ultraviolet B-induced apoptosis [19]. Glycyrrhizic acid ammonium salt is derived from glycyrrhizic acid, which can inhibit hepatocyte apoptosis by regulating the balance of Th cells in the liver [20]. Gypsum can inhibit neurospasm in muscle and bronchus [21]. Bitter Almond has expectorant and antitussive effects. Hence, we hypothesized that MXD may has anti-apoptotic effect in lung tissue, and anti-apoptosis was selected as the entry point for studying the underlying mechanism of MXD.

In the current study, PM2.5-stimulation caused apoptosis both *in vivo* and *in vitro*. After the PM2.5 stimulation, the rats showed symptoms like shortness of breath and nasal cavity effusion. Moreover, TUNEL results also showed a large area of positive cells appeared in lung tissue induced by PM2.5. Furthermore, HE staining in PM2.5-stimulated group shows a serious injury in lung tissue. *In vitro*, after PM2.5 stimulation, the MTT result showed the cell viability was significantly decreased, and Hoechst staining result also showed a large number of apoptotic cells.

In the present study, we investigated whether MXD could display the anti-apoptotic effect as we expected both *in vivo* and *in vitro*. Result of HE staining demonstrated that treatment of MXD could decrease the histopathological lesions’ scores effectively after stimulation. The reduction in TUNEL positive cells revealed that treatment of MXD significantly decreased the apoptotic rate in rat lung tissue. To verify the capability of MXD in regulating cell apoptosis after PM2.5 exposure *in vitro*, we evaluated the therapeutic effect of MXD-medicated serum in A549 cells. The results of the MTT assay in A549 cells indicated that MXD-medicated serum could reduce the cell death rate significantly after PM2.5 stimulation. Moreover, the result of Hoechst staining showed that the level of cell apoptosis was decreased after MXD-medicated serum incubation. MXD forcefully prevented the pulmonary cell apoptosis both *in vivo* and *in vitro*, revealing that MXD is an effective drug for protecting lungs from the stimulation of PM2.5.

There are two primary mechanisms that mediate apoptosis, the internal pathway and the external death receptor pathway [22]. Among them, the intrinsic pathway, which mediated by mitochondria, plays a central role in the regulation of apoptosis. When cells are stimulated by apoptosis signals, the permeability of mitochondrial outer membrane is changed [23]. In the meantime, apoptosis factors such as Cytc are released into the cytoplasm [24]. Mitochondria is mainly regulated by Bcl-2 family [25]. There are two proteins (Bcl-2 and Bax) of different functions in Bcl-2 family. Bcl-2 is an anti-apoptotic protein, which can maintain the mitochondrial integrity by intercepting Cytc [26,27]. Nevertheless, Bax is a pro-apoptotic protein, which can accelerate the release and delivery of Cytc [28,29]. The Bax/Bcl-2 ratio is also widely used to evaluate the level of apoptosis [30]. Caspase-3 is a potent and common effector of apoptosis. C-caspase-3 (the activated form of Caspase-3) triggers the final process of apoptosis and mediates cell destruction [31], and inhibiting C-caspase3 could block apoptosis [32]. Thus, Cytc and C-caspase-3 can reflect the apoptosis. In the present study, PM2.5 could induce the release of Cytc, activate caspase-3, increase the expression of Bax and decrease the expression of Bcl-2. Our results revealed that administration of MXD could decrease the expression of Cytc and C-caspase-3 in PM2.5-stimulated rat lung. Moreover, our data showed that MXD could reciprocally regulate the expression of Bcl-2 and Bax, proving the anti-apoptotic effects of MXD.

In order to further explore the potential signaling pathways related to the anti-apoptotic effects of MXD, we determined the activation of Akt signaling pathway both *in vivo* and *in vitro*. As an upstream kinase of apoptosis, Akt (also known as protein kinase B, PKB) is important in the regulation of cell survival/apoptosis, proliferation and angiogenesis [33,34]. Several reports have observed that Akt is frequently activated among lung injury in previous years, initiating a series of anti-apoptotic signals [35–37]. Accumulating evidence demonstrates that activation of Akt phosphorylation may regulate apoptosis [38–40]. Activation of PI3K/Akt signaling pathway plays an important role in inhibiting apoptosis [41]. It has been demonstrated that the downstream mTOR plays a critical role in airway inflammation and airway hyperreactivity in asthma [42]. The Akt/mTOR pathway can activate p70S6k to modulate cell growth, migration and apoptosis in various cells [43]. Hence, the activation of Akt/mTOR/p70S6K was examined by Western blotting. In the present study, MXD significantly enhanced the expression of p-AKT (S473), p-mTOR (Ser^2448^) and p-p70S6K (Thr^389^) *in vivo* and *in vitro*, indicating that MXD may exert anti-apoptotic effect against PM2.5-induced lung injury via activating the Akt/mTOR/p70S6K signaling pathway.

## Conclusion

Collectively, MXD could prevent cell apoptosis in a dose‐dependent manner via regulating Bcl-2 and Bax expression through the Akt/mTOR/p70S6K pathway. Results of the present study indicate that MXD might be used as an effective agent for the prevention of PM2.5-induced lung injury.

## Supplementary Material

Supplementary Figure S1

Supplementary Materials

## Abbreviations

Akt/PKB: protein kinase B
Bax: Bcl2-associated x protein
Bcl-2: B-cell lymphoma 2
Cytc: cytochrome *c*
C-caspase-3: cleaved-caspase-3
DMEM: Dulbecco’s modified Eagle’s medium
HE: Hematoxylin and Eosin
LSD: least significant difference
MTT: 3-(4,5-dimethylthiazol-2-yl)-2,5-diphenyltetrazolium bromide
MXD: Ma xing shi gan decoction
p70S6K: p70S6 kinase
TUNEL: terminal-deoxynucleotidyl transferase-mediated nick-end labeling
UPLC-MS/MS: ultra-performance liquid chromatography-tandem mass spectrometry/mass spectrometry
