# Supplementary material for: Ma xing shi gan decoction eliminates PM2.5-induced lung injury by reducing pulmonary cell apoptosis through Akt/mTOR/p70S6K pathway in rats
Source: Biosci Rep. 2020 Jul 9;40(7):BSR20193738. doi: 10.1042/BSR20193738 (PMC7350893; doi:10.1042/BSR20193738)
Supplement: Supplementary Figure S1 [file BSR-2019-3738_supp.pdf]

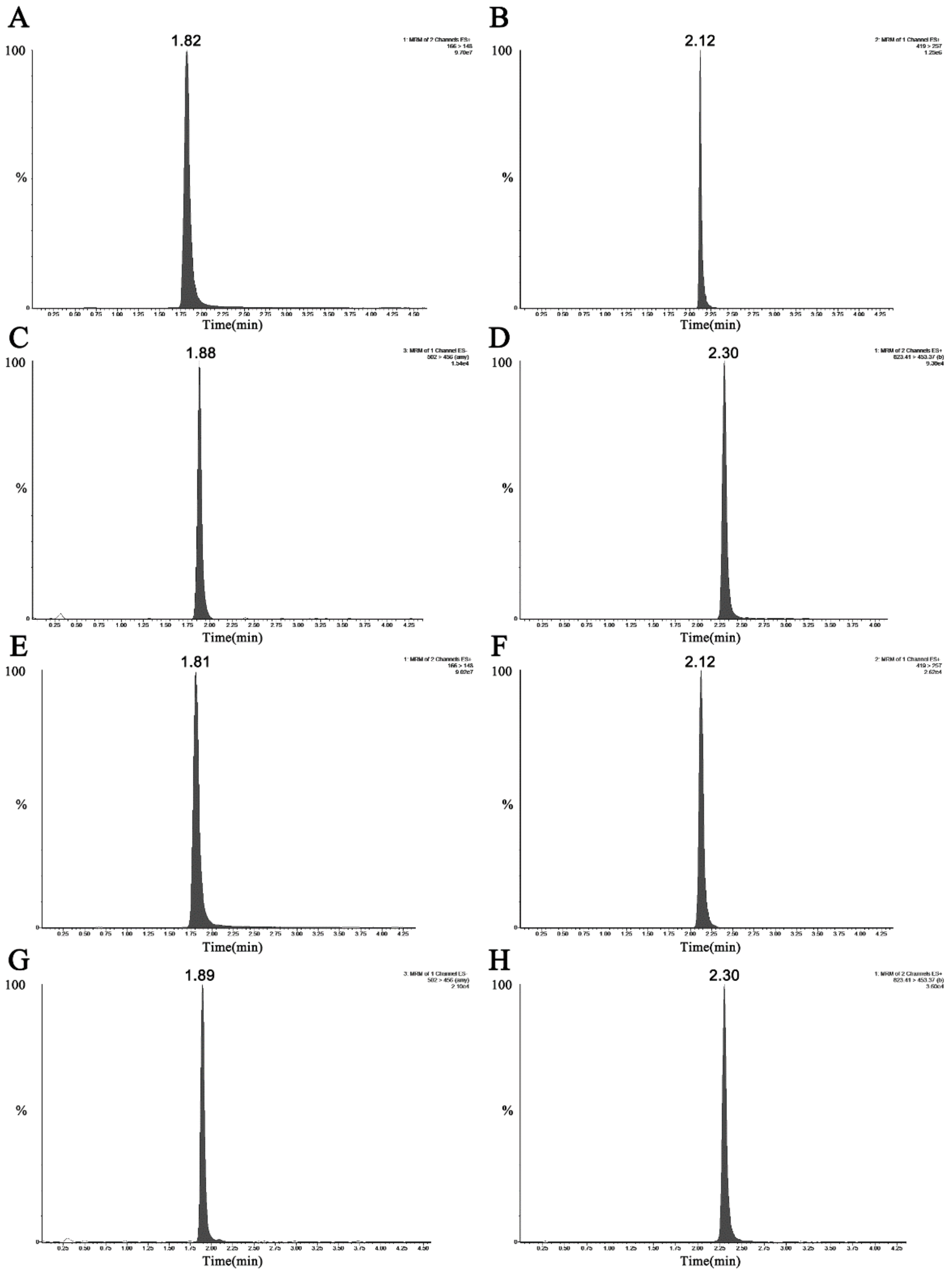

Figure S1. Representative chromatograms of the five compounds in reference standard solutionandmedicated serum. (A) Representative chromatograms of ephedrine (500 ng/mL) and pseudoephedrine (500 ng/mL) in standard solution. (B) Representative chromatogram of liquiritin (500 ng/mL) in standard solution. (C) Representative chromatogram of glycyrrhizic acid (500 ng/mL) in standard solution. (D) Representative chromatogram of amygdalin (500 ng/mL) in standard solution.(E) Representative chromatograms of ephedrineand pseudoephedrinein medicatedserum. (F) Representative chromatogram of liquiritinin medicatedserum. (G) Representative chromatogram of glycyrrhizic acidin medicatedserum. (H) Representative chromatogram of amygdalinin medicatedserum.
