# Supplementary material for: Ma xing shi gan decoction eliminates PM2.5-induced lung injury by reducing pulmonary cell apoptosis through Akt/mTOR/p70S6K pathway in rats
Source: Biosci Rep. 2020 Jul 9;40(7):BSR20193738. doi: 10.1042/BSR20193738 (PMC7350893; doi:10.1042/BSR20193738)
Supplement: Supplementary Materials [file BSR-2019-3738_supp1.pdf]

Supplementary materials

Ma xingshigan Decoction (MXD) eliminates PM2.5-induced lung injury by reducing pulmonary cell apoptosis through Akt/mTOR/p70S6K pathway in rats

Yefang Wang [1a], Bo Zhao [2a*], Yuxiang Fei [2], Qiyang Yin [2], Jianping Zhu [2], Guanghui Ren[2], Bowen Wang[2], Weirong Fang [2*], Yunman Li [2*]

Department of Paediatrics, Nanjing Integrated Traditional Chinese and Western MedicineHospital, Nanjing 210014, PR China.

[2] State Key Laboratory of Natural Medicines, School of Basic Medicine and Clinical Pharmacy, China Pharmaceutical University, Nanjing 210009, PR China

* Corresponding author1: Bo Zhao. E-mail: 532045956@qq.com；

* Corresponding author2: Weirong Fang. E-mail: weirongfang@163.com;

* Corresponding author 3: Yunman Li. E-mail: yunmanlicpu@163.com.

[a]These twoauthorscontributed equally to this study.

Analysis of the medicated serum

After collecting the medicated serum, 250 μL precipitant (Acetonitrile/Methanol, v/v, 1:1) was addedto 50 μL rat plasma sample. The mixture was vortex-mixed for 10 min, centrifuged at 8℃ for 10 min at 15000 rpm, and 2 μL of the supernatant was injected into a column for analysis.

The quantification of ephedrine (and pseudoephedrine), liquiritin, glycyrrhizic acid and amygdalinwas conducted using external standard method with a UPLC-MS/MS system. The UPLC separation was performed at 50 °C using an Acquity UPLC HSS T3 (2.1×30 mm, 1.8 μm) column. Mobile phase solvent A was consisted of aqueous phase of ammonium acetate in concentration 5 mmol/L, and mobile phase solvent B was pure methanol. Positive ionization electrospray MS was employed for ephedrine (and pseudoephedrine), liquiritin and glycyrrhizic acid.Negative ionization electrospray MS was employed for amygdalin.Theinjection volume was 2 μL, and gradient elution conditions are shown in Table S1.The optimal instrument conditions, precursor-product ion combination of m/z for each ingredient were shown in Table S2.

Table S1. Elution condition for UPLC-MS/MS analysis of medicated serum

Time (min)	MP A%	MP B%	Flow rate (mL/min)
Initial	96.00	4.00	
1.00	96.00	4.00	
3.50	30.00	70.00	0.500
4.50	30.00	70.00	
4.60	96.00	4.00	
5.00	96.00	4.00	

Table S2. Optimal instrument conditions

Ingredient	Cone (V):	Collision (eV)	Combination of m/z
Ephedrine and Pseudoephedrine	30	12	166.00→148.00
Liquiritin	42	8	419.00→257.00
Glycyrrhizic acid	42	8	823.41→453.37
Amygdalin	30	12	502.00→456.00

Quantification of mainingredientinthe medicated serum

The representative chromatograms of the five compounds in reference standard solution and medicated serum were shown in Figure S1.The concentration of main ingredients of interest were shown in Table S3.

Table S3. The concentration of main ingredients in medicated serum

Ingredient	Concentration (ng/mL)
Ephedrine and pseudoephedrine	925.8
Liquiritin	13.6
Glycyrrhizic acid	203.4
Amygdalin	519.3
